# Central Nervous System Cryptococcal Infections in Non-HIV Infected Patients

**DOI:** 10.3390/jof5030071

**Published:** 2019-08-02

**Authors:** Justin Beardsley, Tania C. Sorrell, Sharon C.-A. Chen

**Affiliations:** 1 Marie Bashir Institute for Infectious Diseases and Biosecurity, The University of Sydney, Sydney 2145, Australia; 2 Westmead Institute for Medical Research, Westmead, Sydney 2145, Australia; 3 Centre for Infectious Diseases and Microbiology Laboratory Services, Institute of Clinical Pathology and Medical Research, NSW Health Pathology, Westmead Hospital and the Marie Bashir Institute for Infectious Diseases and Biosecurity, The University of Sydney, Sydney 2145, Australia

**Keywords:** Cryptococcosis, *Cryptococcus neoformans*, *Cryptococcus gattii*, central nervous system, meningo-encephalitis, cerebral infection, HIV-negative patients, epidemiology, antifungal therapy

## Abstract

Central nervous system (CNS) cryptococcosis in non-HIV infected patients affects solid organ transplant (SOT) recipients, patients with malignancy, rheumatic disorders, other immunosuppressive conditions and immunocompetent hosts. More recently described risks include the use of newer biologicals and recreational intravenous drug use. Disease is caused by *Cryptococcus neoformans* and *Cryptococcus gattii* species complex; *C. gattii* is endemic in several geographic regions and has caused outbreaks in North America. Major virulence determinants are the polysaccharide capsule, melanin and several ‘invasins’. Cryptococcal plb1, laccase and urease are essential for dissemination from lung to CNS and crossing the blood–brain barrier. Meningo-encephalitis is common but intracerebral infection or hydrocephalus also occur, and are relatively frequent in *C. gattii* infection. Complications include neurologic deficits, raised intracranial pressure (ICP) and disseminated disease. Diagnosis relies on culture, phenotypic identification methods, and cryptococcal antigen detection. Molecular methods can assist. Preferred induction antifungal therapy is a lipid amphotericin B formulation (amphotericin B deoxycholate may be used in non-transplant patients) plus 5-flucytosine for 2–6 weeks depending on host type followed by consolidation/maintenance therapy with fluconazole for 12 months or longer. Control of raised ICP is essential. Clinicians should be vigilant for immune reconstitution inflammatory syndrome.

## 1. Introduction

Cryptococcosis is an infection of global importance with significant attributable mortality. Disease affecting the central nervous system (CNS), manifesting as meningo-encephalitis, is the most common manifestation, with combined CNS and pulmonary infection also important to recognize [1,2]. Worldwide, CNS cryptococcosis is typically associated with HIV infection [1] but in higher-income countries it is increasingly recognized in patients without HIV/AIDS [3,4,5,6,7]. Particular at-risk patient groups include solid organ transplant (SOT) recipients, patients with rheumatic diseases, and those receiving immunosuppressive therapies. Notably, disease also occurs in otherwise immunologically competent hosts.

Studies have demonstrated important differences in epidemiology, risk factors for infection, clinical complications and approaches to therapy between HIV-infected and non-HIV-infected patients, and within subgroups of the latter [3,7]. In addition, these features also differ according to which of the two main pathogenic groups of *Cryptococcus* cause the infection—*Cryptococcus neoformans* or *Cryptococcus gattii* [3,7,8]. This article focuses on CNS cryptococcosis in non-HIV-infected patients including key insights into infection caused by *C. gattii.* A key message is that HIV-negative patients may require a longer duration of induction, and total, therapy, depending on both host and pathogen factors. More recently described risks include the use of newer biologicals and recreational intravenous drug use.

## 2. Microbiology: The Pathogens

The genus *Cryptococcus* comprises over 30 yeast-like fungal species which are ubiquitous in the environment. The majority of human infection is however, caused by only two species complexes, *C. neoformans* and *C. gattii*.

The taxonomy of *Cryptococcus* is controversial, and evolving based on molecular techniques. These two classic ‘species’ have been divided (into at least two species within *C. neoformans* complex and five within the *C. gattii* complex [9], summarized in Table 1) [9,10]. These molecular divisions allow a better understanding of the ecology and virulence of *Cryptococcus*, yet the need to distinguish species to this level in the clinical context is unclear. A detailed description of the taxonomic debate is beyond the scope of this review in which, for pragmatic reasons, we use the terminology *C. neoformans* species complex and *C. gattii* species complex.

Cryptococcal genotype distribution varies by geographical location (Table 1). *C. gattii* species complex genotype VGI is the commonest amongst isolates from Australia and Papua New Guinea, and only sporadically isolated in Southern USA and Mexico and Asia. Isolates from the *C. gattii* outbreak in Canada and the USA (see **Epidemiology Section** below) are of the genotype VGII, with three subtypes [10,11]. Genotype VGIV is rare outside Africa whilst in Asia and Mexico, there is a broad distribution of genotypes. With regards to *C. neoformans* complex, over 90% of infections are due to *C. neoformans* var. *grubii* (VNI-III). This has a worldwide distribution whilst *C. neoformans* var. *neoformans* (VNIV) has mostly been reported from Europe [2,3].

## 3. Epidemiology

An increasing number of studies have focused on the clinical epidemiology and outcomes of cryptococcosis in persons without HIV. The main patient groups are SOT recipients, those with other immunodeficiency disorders, rheumatic disorders, malignancy, diabetes mellitus, cirrhosis and hepatitis B virus infection [3,6,7,12,13,14,15,16]. The results of several published cohorts with more than 20 cases are presented in Table 2.

### 3.1. Transplant Recipients

Many SOT patients require relatively long term and moderately intense immune suppression. This places them at higher risk for invasive fungal infections, with cryptococcosis the third most common infection, affecting approximately 7–10% of such recipients [3,15,16]. Incidence is generally reported as being higher in kidney and heart transplant recipients [16,17] although one US study identified lung transplant recipients as having the highest risk [18]. The US Transplant-Associated Infection Surveillance Network (TRANSNET) study estimated that amongst SOT recipients, the lifetime incidence of cryptococcosis was 12%, and 12-month cumulative incidence was 0.2%, with 90-day and 12-month mortality rates of 15 and 27%, respectively [16]. However, mortality can approach 50% in patients with meningo-encephalitis [19,20]. A recent US study identified cryptococcosis in 0.37% of SOT adults. Of note, despite increasing numbers of transplants, there has been little change in the frequency of cryptococcal disease over the last two decades [5]. The highest proportion of cryptococcosis in SOT recipients has been reported from the USA (18–42%) [17,21,22] with lower proportions in Australia (5%) [7]. The incidence reported in Asia (<1%) is very low [14,23,24,25], likely reflecting differences in transplant practices.

The median time to onset of cryptococcosis is about 20 months after SOT with a significant proportion presenting after 3 years. Time to disease varies by type of transplant. George et al. noted an overall median time to diagnosis of 464 days (range 4–2393) but with a median time of 191 days for lung transplant recipients compared with 200 days and 616 days for liver and kidney transplant recipients, respectively. Very early disease (<30 days) occurred more frequently in liver and lung recipients [18]. This early onset may be related to unrecognized infection pre-transplantation or donor-derived infection. The latter is very uncommon but can present with disseminated disease [26,27]. In this instance, the key is to ascertain the status of other recipients of other organs from the same donor. Screening of cryptococcosis in the donor population e.g., by the serum cryptococcal antigen test has yet to be studied and is not recommended routinely but clinicians should be alert to the possibility of infection in a donor with unexplained lung and/or CNS disease.

Male sex has been considered a risk factor for cryptococcosis in this setting. Other risks for all forms of cryptococcosis are corticosteroid use (all SOTs), hepatic failure or cirrhosis (or liver transplantation) and alemtuzumab therapy [28,29]. Of note, calcineurin inhibitor use is not so strongly associated with central nervous system (CNS) involvement; instead a propensity to cause skin, soft tissue and bone involvement has been reported [15,30]—see **Clinical Features Section** below.

The majority of cases of cryptococcosis in SOT recipients are caused by *C. neoformans* species complex [3,30]. Although overall uncommon, *C. gattii* infection has been well described in the setting of the *C. gattii* outbreak in the USA [31,32] (see later). In a more recent study of 138 HIV-uninfected individuals, only five were confirmed to have *C. gattii* infection. A possible explanation for the apparent skew towards *C. neoformans* (vs. *C. gattii*) could be that laboratories involved in the study did not identify isolates to distinguish between the two species complexes. However, in Australia where most laboratories do differentiate between the two, *C. gattii* remains a rare cause of cryptococcosis in SOT recipients [33].

A question often asked is whether patients who develop cryptococcosis whilst waiting for an organ can still undergo transplantation. Consensus opinion indicates that this is generally safe if infection is under good control, with resolution of clinical features and negative culture results [34,35]. The optimal timing remains uncertain and large studies are required to answer this question.

### 3.2. Non-Transplant Patients

This relatively heterogeneous population of patients comprises those with (i) classical (and significant) immune impairment, (ii) diverse chronic predisposing factors such as end stage organ disease and (iii) apparently immunocompetent hosts with no known predisposing conditions. Such broad host groups make it challenging to produce generic management guidelines. Another important factor is geography - in certain regions, *C. gattii* species complex may account for up to one third of infections in apparently healthy hosts [7]. Knowledge of local epidemiology is essential to inform clinical practice.

#### 3.2.1. Immunocompromised Patients

These include individuals with malignancy receiving chemotherapy, primary or acquired immunodeficincy disorders, rheumatic disorders, and those receiving significant immune suppression for other reasons (including corticosteroids, biologicals such as tumour necrosis factor (TNF)-α inhibitors and other immunomodulatory agents such as tyrosine kinase inhibitors, notably ibrutinib). As with SOT recipients, the proportion of total infections occurring in for these patient groups varies between centres and geographic regions (Table 2).

Underlying malignancy is increasingly recognised as a predisposing factor for cryptococcosis (Table 2). An Australian population-based study in the late 1990s showed cancer in 9.1% of all cryptococcosis patients, and 16% of HIV-uninfected patients [7]. Most of these had leukemia or lymphoma. This pattern was also seen in subsequent studies, showing malignancy in 17–19% of cases with the majority of these malignancies being haematological [6,16,17,22]. In Asia, malignancy as a risk factor was very uncommon in China and Vietnam (<1%) [14,23,25] but reached 16–28.6% elsewhere (Table 2) [24,36,37] (interestingly, mostly solid tumours). Of note, in a small study of haematology patients with cryptococcosis (*n* = 33), CNS disease was more frequent in patients with non-malignant haematological conditions (64%) than those with haematological malignancies (36%) [38].

Patients with underlying rheumatic disorders receiving immunosuppressive therapy have increased risk for cryptococcosis. A review of the literature indicates that the majority of reports focus on patients with systemic lupus erythematosus (SLE) or rheumatoid arthritis (RA). Of 164 HIV-negative patients in one study, 21 had rheumatic disorders (mostly SLE), with 23.2% receiving corticosteroids [14] (Table 2). In two US studies, rheumatic disorders comprised 9.2–15.9% of the study cohort (again, mainly SLE) [6,17] and in Thailand, 16% [24]. A systematic review of SLE patients identified a prevalence of cryptococcal meningitis of 0.5%, where almost 40% of patients had markers of active lupus, and where prednisone use was associated with higher mortality [39]. In rheumatoid arthritis (RA) patients, both disease-related and iatrogenic immune dysfunction affect the risk of cryptococcosis, especially corticosteroid and TNFα inhibitor use. Corticosteroids are a well-known risk factor for severe infection in rheumatoid arthritis patients [40]. Since TNFα production is essential in the immune response against cryptococcal infection [41], it is understandable that depleting TNFα with biologicals may facilitate development of cryptococcosis. In one study of 20 RA patients with cryptococcosis, increased risk was seen in patients with chronic kidney disease and those exposed to the anti TNF antibody, adalimumab [42]. In this study, patients receiving anti-TNF antibodies developed disease after 1.5 ± 1.2 years compared with 8.4 ± 5.5 years in patients not receiving them (*p* < 0.001) [42]. Most if not all cases in RA patients are due to *C. neoformans*.

Idiopathic CD4+ lymphopaenia is also a risk factor for both *C. neoformans* and *C. gattii* infection [33,43], as is the presence of anti-GM-CSF autoantibodies [44]. Most notably, the increasing use of the novel oral tyrosine kinase inhibitor, ibrutinib has been associated with increased risk for cryptococcosis, particularly CNS disease [45,46].

#### 3.2.2. Hosts with Chronic Diseases and Immunocompetent Patients

Also heterogeneous, these individuals include those with end-stage liver disease, renal insufficiency, sarcoidosis and diabetes mellitus (Table 2), but also patients who are phenotypically normal with no underlying medical conditions or predisposing factors.

Diabetes mellitus is generally considered to be a risk factor for cryptococcosis. Although an Australian study found no association between the two entities [7] and the frequency of diabetes mellitus in CNS cryptococcosis patients has been reported to be very low [25], this is in contrast to findings from elsewhere. A recent Taiwanese study of HIV-uninfected patients found that those with cryptococcosis were more likely to have diabetes than matched controls (*p* < 0.001) [13]. Diabetes was associated with increased 1 year and overall mortality from cryptococcal meningitis [13]. Li et al. in another study reported a hospital-based prevalence of cryptococcosis in Type II diabetes of 0.21% where infection was associated with poor control of diabetes. Mortality is reported to be 33% [49].

The proportion of patients with cryptococcosis with underlying chronic liver disease and renal insufficiency have also varied between studies [17,21,24,25,36]. Chronic renal failure ranged from 0.1–17% of patients whilst chronic/decompensated liver diseases including cirrhosis and HBV carriage comprised 3.5–30.2% of patients. Patients in these studies had *C. neoformans* complex infections.

CNS cryptococcosis also occurs in otherwise healthy hosts (Table 2). A population based study of cryptococcosis in Australia showed that 109/349 (31.3%) cases occurred in immunocompetent hosts; in this study just over 50% were caused by *C. neoformans* species complex, and the remaining by *C. gattii* [7] The proportion of normal hosts in US surveys varied from 17–22% [17,21,22]; all caused by *C. neoformans*. Prevalence in Asian countries ranges from 7.8–81% [14,25,37]. All are case series of sporadic infection and do not take into account the outbreaks of *C. gattii* cryptococcosis (discussed below). Determining host risk is important as data indicate that clinical features differ between immunocompromised and immunocompetent hosts (see **Clinical Features Section**).

New risks in otherwise immunocompetent hosts include recreational intravenous drug use, which has the potential to cause case clusters [50,51]. Also of note, prolonged influenza virus infection may predispose to cryptococcosis [52], supported by in vivo mice studies which demonstrate reduced phagocytosis and killing of cryptococci in influenza-infected macrophages and adverse outcomes [53].

### 3.3. C. gattii Infection

*C. gattii* characteristically affects apparently immunologically normal hosts [7,33,54,55]. It is endemic in Papua New Guinea (incidence 42.8/million/year) and in Australia, especially the Northern Territory (8.5/million population/year) (summarized in [55]). Host genetic factors, immune status, geography and environmental exposure likely all contribute to risk of cryptococcosis since the incidence in Australia’s aboriginal population is 10.4/million/year (vs. rate of 0.7/million/year in non-indigenous people) even correcting for place of residence; the commonest molecular genotype was VGI and mortality ranged between 0–15% [7]. Sporadic cases have also occurred in southern USA and South America [55].

The notion that *C. gattii* was restricted to tropical and subtropical areas changed with the advent of outbreaks of infection, beginning in 1999, in the temperate areas of British Columbia, Canada and the Pacific Northwest USA [11,32,54]. It is accepted that the outbreak strains were introduced from South America [56] and the outbreak is continuing. The rate of disease in British Columbia at the peak of the outbreak (1999–2007) was 0.3–0.5 per 100,000 population (http://www.bccdc.ca/resrouce). MacDougall et al. established that in the outbreak setting, risk factors for infection included age of >50 years, smoking, corticosteroid use in the preceding 3 months, HIV infection, malignancy and chronic lung disease [32]. However, many patients had respiratory infection only. In the US Pacific Northwest clusters, CNS disease was less common, and only one third had underlying predisposing conditions (ummarized in [3]). Because many of those infected were older, it is difficult to compare clinical outcomes [54].

Of interest, the outbreak in North America resulted largely from clonal expansion of genotype VGII, specifically three subtypes—VGIIa, VGIIb and VGIIc. In British Columbia, VGIIa accounted for 86.3% cases, with other subtypes rare or absent; in contrast the distribution of subtypes in the US was VGIIa (50%), VGIIc (32%) and VGIIb (10%) [57]. The dominant VGIIa is more virulent, as manifest by a high intracellular proliferation rate within macrophages and plasticity of its mitochondrial morphotype (meaning it can resist oxidative stress within the macrophage) [58,59]. It also manifests a ‘division of labor’, whereby hardy but slowly reproducing individuals signal others to proliferate more rapidly [59] (see also **Pathogenesis Section** below). VGIIc is phenotypically similar to VGIIa but has been isolated only in the USA [58].

## 4. Pathogenesis

The pathogenesis of CNS infection in HIV-negative individuals is similar to that in HIV patients.

### 4.1. Cryptococcal Virulence Determinants

*C. neoformans* and *C gattii* species complexes express the same suite of major virulence determinants. These include the polysaccharide capsule (important in evasion and suppression of the host immune response), melanin (protects cryptococci against oxidative stress), the invasins, phospholipase B (Plb1) and urease, and antioxidants, superoxide dismutase (SODp1) and trehalose [55]. In animal models of *C. neoformans* infection, Plb1 and laccase are essential for egress of cryptococci from the lung and dissemination to the CNS [60,61] whereas Plb1 and urease are required for passage across the blood–brain barrier [61,62]. Genomic and transcriptomic studies have revealed enormous complexity in expression of an individual ‘virulence phenotype’ with involvement of multiple genes, transcription factors, and networks of signaling pathways in addition to virulence determinants [55,63,64,65].

### 4.2. Pathogenesis of Cerebral Cryptococcosis

The pathogenesis of CNS cryptococcosis remains poorly understood. Passage across the cortical microvasculature as free cells and within mononuclear phagocytes and possibly neutrophils (Trojan Horse mechanism) has been demonstrated in vitro, as well as in animal models [61,62,66]. These cells promote establishment of cerebral cryptococcosis, which remains rudimentary in their absence [61,66,67]. The early innate immune response in neurocryptococcosis comprises myeloid cells and lymphocytes recruited from the blood into the perivascular space, and it is proposed that cerebral cryptococcosis arises via a two-step process. First phagocyte-dependent transport into the perivascular space (via transcellular endothelial migration [68,69,70,71] or paracellular passage), then release of cryptococci by non-lytic exocytosis [72,73] followed and penetration of the glia limitans. Cryptococci may disrupt the glia via local release of cryptococcal metalloprotease, Mpr1 [74], urease [62], phospholipase B1 [61] and enzymes in secreted microvesicles [75] which have been implicated in cryptococcal penetration of the cerebral microvascular endothelium.

### 4.3. Meningitis

It has been postulated that meningitis develops following rupture of cryptococcomas into the subarachnoid space [76,77]. However, the perivascular space may provide an alternative route. MRI scans in human infection reveal expanded perivascular spaces [78]. The perivascular space [79] is only separated from the subarachnoid space by the pia mater [79,80], adding to the plausibility of this route.

## 5. Clinical Features and Complications

It is difficult to summarize the clinical features of non-HIV associated cryptococcal CNS infections because of the heterogeneity of the patient population (as in **Epidemiology Section**). The balance of patients at the two ends of this spectrum, i.e., those who appear completely healthy, to those who have received significant amounts of immunosuppressive medication, varies according to setting, based on differences in prevalence of underlying diseases and access to immunosuppressive medications (Table 2).

### 5.1. Clinical Presentation

Disease phenotype differs according to immune function, in what has been described as a parabola effect [81]. Those with deficient CD4+ T-cell responses fail to control fungal growth, and end up with high fungal burden but pauci-inflammatory disease (as in HIV-associated disease) [82,83]. Those with over-active CD4+ T-cells, dysregulated macrophages, and Th1 skewed inflammatory responses clear fungi effectively but suffer tissue damage, consistent with clinical experience and as demonstrated in mouse models [84]. This parabola may partly explain the disappointing outcomes from immune-therapeutic interventions (in HIV patients) to dampen or stimulate the immune response [85].

Nonetheless, there are some common features-cryptococcal meningitis, whatever the underlying condition, presents with classical meningitic features, including headache, fever, and vomiting for the majority of patients (see Table 2). However, diagnosis may be delayed in non-HIV patients, likely due to low clinical suspicion and insidious onset. Focal neurological deficits are major features of non-HIV associated cryptococcal meningitis [17,33].

Clinical features are well-described in SOT recipients where presentation may be atypical, and where there have been differences in presentation depending on SOT type. Disease may be disseminated, with multiple body sites involved simultaneously. *Cryptococcus* can infect any body site, notably skin and soft tissues, muscles, bones, eyes, genitourinary and gastrointestinal tracts, thyroid and adrenal glands (summarized in [15]). In one cohort of SOT recipients, involvement of the CNS was followed in frequency by fungemia, lung, and localized disease [86]. In CNS infection, meningitis, parenchymal abscesses (or cryptococcomas) and hydrocephalus were observed in 14%, 12% and 4% of patients, respectively. Parenchymal lesions seem to confer a higher mortality [87].

Osawa et al. showed that the predictors of CNS disease include altered mental status, late onset disease (>24 months), serum cryptococcal antigen greater than 1:64 and fungemia [88]. In this study, frequently encountered symptoms were headache, altered mental status and visual symptoms. In liver transplant patients, liver failure was associated with cryptococcal meningitis mortality [89].

### 5.2. C.gattii Compared with C. neoformans Infections

Most differences in clinical features between *C. neoformans* and *C. gattii* infection may be explained by differences in host population. The issues related to heterogeneity of hosts described above also applies when trying to compare clinical outcomes between patients infected with *C. neoformans* and *C. gattii*. For example, it is widely accepted that *C. gattii* is more likely than *C. neoformans* to cause lung disease. Whilst one review of the *C. gattii* cases from British Columbia found that *C. gattii* has a predilection for causing lung disease, with over 75% presenting with lung infection and only 20% with CNS infection, the authors note that this could just mean that the dissemination to the brain is less likely in otherwise healthy, or minimally immune-compromised, hosts [54]. Furthermore, in contrast to this finding, reports from Australia show that *C. gattii* causes CNS disease in 85% of cases, with lung involvement (solely or concomitantly) in 60%, although lung cryptococcomas arose more often with *C. gattii* regardless of host immune status [33]. Chau et al. showed no statistically significant differences in clinical presentation of *C. gattii* and *C. neoformans* for those without HIV-infection. They described chest X-ray abnormalities more often in *C. neoformans* (32%) vs. *C. gattii* (20%) infection, although numbers of *C. gattii* infection were small and the difference was not statistically significant [25].

### 5.3. Complications

Some of the more devastating complications of cryptococcal meningitis and CNS disease include raised intracranial pressure, cryptococcomas, and cerebral infarction. Indeed, intracranial pressure >20 cm of water can occur in a substantive proportion of patients (Table 2) and requires careful management, including serial lumbar puncture or the insertion of a shunt [90,91]. In a series with relatively low mortality of 15%, the authors attributed the good outcomes to aggressive neurosurgical interventions in cases of raised intracranial pressure [33]. One 2010 review identified 17 cases of cryptococcoma in patients with no history of immunosuppression [92] and an earlier study from Australia showed that cryptococcoma was more common in HIV-negative patients [7]. CNS lesions of >3 cm in size often require surgery [90]. Infarction, possibly related to vasculitis or local pressure effects, can also occur [93]. Magnetic resonance imaging (MRI)-based studies have reported incidence rates of infarction of 18–44% [94,95]; although both studies included patients with underlying cerebrovascular disease and/or diabetes, infarction is probably under-recognized.

Another complication, which seems to occur more frequently in *C. gattii* infection (and therefore non-HIV associated infection) is visual impairment including blindness, which occurs in up to 40% of patients, perhaps suggesting some tropism of *C. gattii* for visual centres [33,96].

Immune reconstitution inflammatory syndrome (IRIS) is well-described in HIV-associated cryptococcal meningitis [97,98]. Despite microbiological control, patients may develop meningitis, lymphadenitis, pneumonitis, and cryptococcomas. The pathophysiology of IRIS in the iatrogenically immunosuppressed population is analogous to what happens in HIV/AIDS, except that immune reconstitution arises from withdrawal of medication rather than administration of anti-retrovirals [3]. However, a type of IRIS can also occur in apparently healthy patients taking no immunosuppressive medications. In a detailed series of 86 patients with *C. gattii* infection, IRIS occurred in seven patients (8%), and five (6%) of those had no identified immunosuppression [33]. Despite clearing infection with treatment, patients display a paradoxical clinical exacerbation caused by an exuberant immune response, characterized by infiltration of activated T-lymphocytes, destructive cytokine patterns, and ineffective macrophages [99]. This may result from Cryptococcus losing its ability to evade immune surveillance, and exposing its antigens, as it dies.

## 6. Diagnosis

Any sign of CNS infection in an individual at risk for cryptococcosis should prompt diagnostic investigations. In addition to microbiological diagnosis, imaging of the CNS is indicated. Computerized tomographic (CT) scans of the chest, which often provide an early diagnostic clue, are necessary to exclude pulmonary infection and to define disease extent. In areas endemic for *C. gattii*, circumscribed cryptococcomas, both in patients with lung infection only (83%), and in those with combined lung and CNS disease (77%) [7,33] may be seen. In the BC outbreak, 75% of patients had pulmonary nodules [54] In *C. neoformans* infection, alveolar and interstitial pulmonary infiltrates account for 14–17% of chest abnormalities and occur more often (26–70%) in immunocompromised hosts [7,54].

### 6.1. Neurological Imaging

Imaging of the brain is essential to exclude parenchymal involvement and other complications. Single or multiple cryptococcomas with or without contrast enhancement and edema, hydrocephalus and areas of infarction may be seen (see **Complications Section** above). These abnormalities can occur in both *C. neoformans* and *C. gattii* infections. In one study, cryptococcomas, most commonly in the cerebellum, basal ganglia and thalamus, were observed in cerebral CT scans in 33–58% of cases and were more frequent in *C. gattii* infection (24/30 cases) compared with *C. neoformans* (18/81 cases, *p* < 0.001) [7]. Obstructive hydrocephalus may be seen in 17–36% cases [7,100,101]. Conversely, dilatation of the perivascular spaces, due to accumulation of organisms and capsular material within, is more easily observed on MRI, being reported in almost 50% of cases of HIV-associated CNS cryptococcosis due to *C. neoformans*. These changes were typically in the basal ganglia [102] and it is assumed they are also present in *C. gattii* infection. MRI is preferred over CT for its greater sensitivity in detecting small mass lesions and basilar meningeal enhancement [102].

### 6.2. Microbiological Diagnosis

Microbiological diagnosis of CNS cryptococcosis in not usually difficult as culture of both *C. neoformans* and *C. gattii* species complex from CSF or from brain tissue is straightforward. Where microbiology laboratories do not identify *Cryptococcus* to the species complex level, clinicians should be aware that a report of “*C. neoformans*” indicates both species complex.

#### 6.2.1. Culture and Histopathology

The majority of CNS infection can be diagnosed by visualizing or culturing the organism from clinical specimens and/or by a positive serum, or CSF, cryptococcal polysaccharide antigen (CRAG) test. As CRAG detection does not allow speciation of cryptococci, culture is essential for definitive diagnosis. CSF examination is mandatory unless there is a contraindication to lumbar puncture, and brain biopsy may be useful where indicated. Other clinical specimens such as bronchoalveolar lavage (BAL) fluid, sputum, blood, and aspirate/biopsy sections of affected sites should also be cultured to aid diagnosis and determine disease extent. Culture is similarly sensitive in healthy and immunocompromised hosts.

Histopathological examination of biopsy specimens as appropriate is also useful for diagnosis. Grocott methenamine silver (GMS) and periodic acid–Schiff stains will show yeast forms consistent with *Cryptococcus*. However, either mucicarmine or alcian blue, combined with the Fontana–Masson stain is superior in detecting cryptococci and more specific than GMS and stains such as calcoflour white since it highlights both the cell wall (Fontana–Masson) and mucin-positive capsule (mucicarmine/alcian blue) [103]. The Fontana–Masson stain, which detects melanin, also has the advantage of being able to distinguish rare capsule-deficient strains.

India Ink staining of CSF is valuable for rapid (<5 mins) detection of encapsulated cryptococci (both *C. neoformans* and *C. gattii*). The diagnostic sensitivity for *C. neoformans* has been reported to be lower in HIV-negative patients (30–72%) than in those with AIDS (80%) [104]. In one study, the CSF India ink was positive in 70% of patients with *C. gattii* meningitis [105] and in almost 95% of cases in another [7]. For laboratories not performing India Ink smears, CSF Gram stains have similar sensitivity if cytospin preparations of CSF are examined.

Clinical specimens may be plated onto selective mycological media as well as on standard bacteriological agar. In the majority of cases, use of canavanine glycine-bromothymol blue (CGB) agar is recommended as a simple method to reliably distinguish colonies of *C. gattii* (colonies become blue) and *C. neoformans* (no change in colony colour) based on utilization of glycine and susceptibility to canavanine [106]. Serotyping of *C. gattii* into B or C serotypes is not routinely performed as the Crypto Check kit (Iatron Laboratories, Tokyo, Japan) is no longer available.

Identification of yeasts by matrix-assisted laser desorption ionization-time of flight mass spectrometry (MALDI-TOF MS) is now routine in most clinical laboratories. Both the Bruker (Bruker Daltoniks, GmbH, Germany) and Vitek MS (BioMerieux, Marcy-l’etoile, France) systems have been studied for their ability to identify, and distinguish between *C. neoformans* and *C. gattii.* Where the in vitro diagnostic (IVD) databases in the two commercial systems, are used, distinguishing between the two species complex may not be possible. However, using the respective research only software (RUO), and particularly with complementation with in-house generated spectra, rapid and reliable identification is readily achieved. Furthermore, accurate identification of all the genotypes of *C. neoformans* and *C. gattii* has been reported [107,108,109].

#### 6.2.2. Cryptococcal Antigen

Detection of CRAG in body fluids has high sensitivity and specificity (100%) in the diagnosis of all forms of cryptococcosis. For diagnosing CNS disease, serum CRAG testing has a sensitivity of 94–100%, and CSF CRAG testing has a sensitivity of 87–100%, including for *C. gattii* infection and regardless of HIV status [7,106]. False-negative results in CNS disease are very uncommon, but may occur as a result of a prozone effect (where the amount of antigen in the sample is in excess of the amount of antibody in the assay; this can be overcome by sample dilution) or from rare capsule-deficient strains. False-positive tests are also uncommon [106]. In *C. neoformans* infections, CRAG has been detected in urine and lung fluid but the utility of the CRAG test in specimens other than blood and CSF is uncertain. The two most common test formats used to be the latex agglutination (LA) and enzyme-linked immunoassays (EIAs). These tests increasingly, are superseded by the cryptococcal lateral flow assay (LFA) for CRAG detection. This is because of the LFA format is now established as a point-of-care test to diagnose and screen patients for cryptococcosis, especially in resource-limited regions. It is simple, rapid (10–15 mins), requires little specialized skill, and does not require refrigeration or substantive laboratory infrastructure; costs are USD 1.25–2.50/test. On the other hand, LA is more labor intensive, slower (about 4 hours) and requires cold chain handling [110].

At the time of writing, there are five commercially available CRAG LFAs. The first is the IMMY Immuno-Mycologics, Norman, OK) which is FDA-approved in the US and CE-marked in Europe. This kit has undergone large scale multi-site evaluation globally. Two other LFA formats have had limited validation and none are yet FDA-approved—the Biosynex Cypto PS LFA (Biosynex, Paris, France which is CE marked in Europe), StrongStep (Liming Bio, China)—with sensitivity (78–100%) and specificity (90–100%) in serum/CSF compared with the IMMY LFA [summarized in 109]. The remaining two tests as yet have no published validation data.

The IMMY LFA (Immuno-Mycologics) has enabled a paradigm shift in diagnostics and preventative screening efforts. Its simple “dipstick” format uses monoclonal antibodies directed at glucuronoxylomannan (GXM) to capture CRAG of both *C. neoformans* and *C. gattii,* with both qualitative and semi-quantitative results. Data from studies examining the performance of the LFA on samples from HIV-positive patients with *C. neoformans* infection, indicate sensitivities of 90–99.8% when tested on serum, 70–98% for urine samples and 96–100% for CSF, with specificities of >99% [110,111,112,113]. Results compared with EIA tests showed good agreement and dipstick titers correlated strongly with EIA [112,113]. Of note, the dipstick is only semi-quantitative and titers obtained by LA have not been systematically correlated with those of the dipstick test. Sensitivity and specificity of the LFA in the diagnosis of *C. gattii* infection is likely to be similar to that for *C. neoformans* as has been shown by serial testing of a small number of samples (*n* = 12) from two patients [114].

LFA CRAG screening on blood for HIV-infected persons can detect infection prior to development of cryptococcal meningitis and is now recommended by the WHO [111]. However, whether the LFA has similar utility in screening HIV-negative individuals, and for screening for *C. gattii* infection, is uncertain. Prospective studies are needed to determine the position of the LFA as a screening tool for asymptomatic cryptococcaemia, in high-risk patients.

The utility of serum or CSF CRAG testing in monitoring response to therapy is uncertain. Concordance between titers and clinical or mycological outcomes is often poor. Titers remain elevated for prolonged periods (months to >1 year). CRAG results should therefore be interpreted in conjunction with clinical assessment.

#### 6.2.3. Molecular Diagnostics

Molecular tests can distinguish between *C. neoformans* and *C. gattii* but are seldom required for diagnosis. In the uncommon event that the isolate does not grow or is seen only in histological sections, then molecular methods may be used. These techniques demonstrate good overall sensitivity in identifying both *C. neoformans* and *C. gattii* species complex in clinical specimens, or species complex identification of cultured organisms [106,115]. PCR-based methods, combined with DNA sequencing are preferred. Internal transcribed spacer (ITS)-targeted assays are most frequently used with good clinical utility and can also distinguish between genotypes of the both species complexes [116].

## 7. Antifungal Therapy and Management of Complications

Management of CNS cryptococcosis in non-HIV patients depends on the type of host and often requires a multidisciplinary approach. The involvement of the infectious diseases service was shown in one study to reduce mortality [117]. There are some data that ideal antifungal therapy, specifically duration of therapy, differs for *C. gattii* and *C. neoformans* infection [90,118]. Regardless, treatment is complex and must take into account associated toxicity from antifungal drugs used. Dosing of amphotericin B deoxycholate, 5-flucytosine and the azoles must be adjusted for kidney function. Regular monitoring of renal function, serum electrolytes, hematological parameters and assessment of drug-drug interactions is essential.

Unlike for the HIV/AIDS population where therapy is informed by large scale randomized controlled trials, similar prospective studies are scarce to determine the optimal antifungal regimens in non-HIV infected patients. In general, treatment is guided by recommendations of expert bodies such as the Infectious Diseases Society of America (IDSA), Australia and New Zealand Mycoses Interest Group (ANZMIG) and American Society of transplantation guidelines [90,118,119]. Both the IDSA and ANZMIG guidelines divide treatment strategies according to host risk and these are summarized in Table 3. Similar to HIV-associated cryptococcosis, treatment encompasses induction, consolidation, and maintenance phases of antifungal therapy [90,118,119,120].

### 7.1. Use of Azoles and Azole Resistance

Fluconazole is well-established as the cornerstone antifungal agent in the consolidation and maintenance phases of therapy for cryptococcosis, with its good overall efficacy and favorable safety profile. In general, for both species complex, resistance to this azole is uncommon. A systematic review of 29 studies spanning 1998 to 2017 found a baseline resistance rate of 12% although it remains unclear how this ‘resistance’ was defined [122].

Many studies however, have documented low antifungal MICs against *C. gattii* and *C. neoformans*, which have remained so over time [123]. However, in some countries, *C. gattii* isolates were less susceptible to some antifungal agents. Trilles et al. reported higher geometric mean minimum inhibitory concentrations (MICs) for fluconazole, voriconazole, amphotericin B and 5-flucytosine against *C. gattii* compared with *C. neoformans* [124] and in another study, fluconazole, voriconazole and posaconazole MICs were significantly higher against *C. gattii* [125]. Increased fluconazole MICs have also been reported from the US Pacific Northwest to suggest that the genotypes VGIIa and VGIIc may exhibit increased MICs to fluconazole and voriconazole [126,127]. Whether these higher MICs imply the need for higher doses for therapeutic effect is uncertain.

Itraconazole may exhibit similar effectiveness to fluconazole during consolidation/maintenance therapy at a dose of 600 mg daily, whilst posaconazole and voriconazole also have anti-cryptococcal activity and are alternative options if fluconazole is unable to be administered, or as salvage therapy. Likewise, the newer azole, isavuconazole also has good in vitro activity against *Cryptococcus* [128]. Because there are no clinical MIC breakpoints to assign isolates as ‘resistant’ or susceptible’ to these newer azoles or indeed to fluconazole, it is not possible to determine the prevalence of resistance. Studies correlating MICs with clinical outcomes are needed.

### 7.2. SOT Recipients with C. neoformans Disease

In SOT recipients with CNS (meningitis, cerebral disease) and/or disseminated infection due to *C. neoformans*, 2 weeks of induction therapy with combined lipid formulation of amphotericin B plus 5-flucytosine is recommended. Although amphotericin B deoxycholate can be used if lipid formulations are not available, SOT recipients are at high risk for nephrotoxicity associated with the concurrent use of amphotericin B and calcineurin inhibitors; hence a lipid formulation is strongly preferred [90,118]. In one small prospective study of 75 SOT patients, decreased mortality was shown in those treated with a lipid formulation of amphotericin B (liposomal amphotericin B, or amphotericin B lipid complex); furthermore, mortality did not differ between patients receiving lipid amphotericin B with or without 5-flucytosine [129]. In general, there is more widespread experience with the use liposomal amphotericin B amongst lipid formulations. For patients with meningitis and a positive baseline CSF culture, a repeat lumbar puncture is recommended before stopping induction therapy. If CSF cultures remain positive, then a longer course of therapy is advised (e.g., 4–6 weeks) (See **Duration of Therapy Section** in **Section 7.4** below) [3,90,118]. Consolidation and maintenance therapy is fluconazole (400 to 800 mg for 8 weeks followed by 200–400 mg for up to 12 months) [90,118]. Use of this approach is associated with low risk of relapse [90]. Fluconazole dosing must be appropriate for renal function.

Reduction of immunosuppression to assist with eradication of infection should be undertaken cautiously in a graded fashion to minimize the risk of IRIS [35]. Abrupt reduction of such therapy has been associated with worsening of clinical symptoms and risk of graft loss. If undertaken, the recommended approach is to first gradually reduce the corticosteroids and then to reduce the calcineurin inhibitor dose, and those of other immunosuppressive agents [90].

### 7.3. Non-SOT Recipients with C. neoformans Infection

Treatment recommendations for *C. neoformans* infection for the non-HIV non-transplant population including immunocompetent hosts are also mostly based on results from clinical trials among HIV-infected patients [90]. Three are few recent data regarding the duration of induction antifungal therapy for this group of patients. Most experts favour more than 2 weeks e.g., 4–6 weeks of induction therapy with amphotericin B with or without 5-flucytosine [3,90,118] followed by step down therapy to fluconazole 400–800 mg daily for up to 12 months, depending on clinical response.

Although guidelines recommend the use of amphotericin B deoxycholate as induction therapy in non-SOT recipients, the use of lipid formulations is preferred in many settings because of the lower risk of nephrotoxicity. Amphotericin B-based therapy remains a common option to treat cryptococcosis among HIV-negative patients on renal dialysis. If 5-flucytosine is unavailable, amphotericin B can be given with fluconazole [90,120]. Conversely, if amphotericin B is not available or in cases of amphotericin B-intolerance, fluconazole may be combined with 5-flucytosine. Even though there is some weak evidence showing positive outcomes among HIV-negative patients receiving only fluconazole as induction therapy for cryptococcal meningitis, even high dose fluconazole monotherapy remains an inadequate option to treat CNS disease and is not recommended [130].

Of note, the use of 5-flucytosine in patients with renal impairment, including SOT recipients, (**Section 7.2** above), and those receiving renal replacement therapy should be undertaken with care. Despite appropriate dose reductions, patients on intermittent haemodialysis or continuous renal replacement therapy may exhibit supratherapeutic levels in blood, leading to drug-related toxicity such as leucopaenia and thrombocytopaenia [131]. Regular monitoring of trough blood levels is essential to minimise toxicity.

### 7.4. Duration of Antifungal Therapy for C. neoformans Infection

As above, the standard recommended duration of induction antifungal therapy is 2 weeks among SOT recipients (in the absence of complications, provided a good clinical response, and where immunosuppression can be reduced). Assessment for complications e.g., seizures, focal neurologic deficits, ocular involvement, or cryptococcomas, should be undertaken. If any are found, if the CSF is not sterile at 2 weeks, or if there is severe ongoing immunosuppression a longer duration of induction is recommended (generally 6 weeks) [120].

Following a large randomized controlled trial in HIV-associated cryptococcal meningitis, which showed that one week of amphotericin B and 5-flucytosine for induction had the lowest 10-week mortality and least frequent side-effects, the World Health Organisation has changed its recommendations for patients with HIV/AIDs [132]. Whether a subset of HIV-uninfected individuals with CNS cryptococcosis may benefit from shorter induction times is untested and unknown.

### 7.5. C. gattii Infection

In patients without AIDS, physicians treating *C. gattii* CNS infection favor a 4 to 6 week course of induction therapy with amphotericin B formulation and 5-flucytosine (25 mg/kg every 6 hours) based on clinical experience [33,90,118] and extrapolating from favorable outcomes in AIDS patients [121]. Amphotericin B deoxycholate (0.7–1.0 mg/kg daily) has often been used but if there is renal toxicity, lipid base formulations such as liposomal amphotericin B (3–5 mg/kg daily) are as effective [90]. Regular monitoring of renal function, and blood electrolyte and hematological parameters are essential. Duration of induction therapy is not well defined. In some cases, a longer period of induction therapy is required due to slower response to treatment e.g., in intracerebral infection (see below).

Consolidation therapy with fluconazole 400 mg daily for eight weeks followed by maintenance therapy (200 mg daily for approximately 12 months) is the regimen of choice to eradicate infection, and to prevent relapse [90]. The total duration of antifungal therapy however depends on clinical and mycological responses with many clinicians adopting a more conservative approach, and extending total duration beyond 12 months [133]. Experience with use of itraconazole, voriconazole or posaconazole as alternatives to fluconazole as maintenance therapy in *C. gattii* meningitis is limited [90] (see also **Section 7.1**). Treatment of patients from areas of VGIIa/c endemicity should consider the variable azole susceptibilities, with consideration of alternative agents, as directed by susceptibility testing.

#### Intracerebral Infection

The optimal management of cryptococcomas is not well established. Intracerebral infection with both *C. gattii* and *C. neoformans* has been associated with substantial neurological sequelae, need for more frequent neurosurgical intervention, and a delayed or poor response to therapy, typically attributed to the presence of cerebral cryptococcomas [3,101,134]. Hence, more prolonged therapy, continuing combined amphotericin B-flucytosine induction therapy beyond 4–6 weeks may be required [120,121]. Experience indicates that management of CNS infection should also be guided by the appearance of the cerebral CT scan at presentation as this has been shown to correlate with outcome [133]. Accessible large cryptococcomas with surrounding edema, with or without mass effect, should be considered for early surgical removal to improve response to antifungal drugs. Multiple mass lesions may require prolonged (>6 weeks) induction and eradication (>12–18 months) therapy. Corticosteroids administered as adjunctive therapy can effect good outcomes [135,136] and are recommended if both are present [90]. Since one of the more critical determinants of outcome of cryptococcal meningoencephalitis is control of CSF pressure, CSF pressures should always be measured as part of the lumbar puncture procedure and control of raised ICP achieved by repeated lumbar puncture or by early shunting [90,137]. Symptomatic hydrocephalus requires antifungal therapy plus early relief of ICP by shunting. In the absence of cryptococcomas the antifungal regimen should be as for meningitis.

Of note, reliance on imaging to guide duration of therapy can be deceptive and misleading as intracerebral cryptococcomas persist for prolonged periods (>1–2 years) [33]. Re-imaging is indicated in apparent cases of relapse and may reveal new lesions, enlargement of lesions and/or increased peri-lesional oedema despite effective antifungal therapy. In cases with mycological cure, these features appear to be consistent with sterile arachnoiditis and/or an IRIS-like syndrome, potentially warranting corticosteroid administration (see **Section 7.6**).

Recombinant interferon gamma has been tried as an additional modality in patients with cryptococcomas and/or severe meningitis who were unresponsive to prolonged courses of multiple antifungal drugs. Its contribution to subsequent outcomes is uncertain.

### 7.6. Immune Reconsitution Inflammatory Syndrome

In CNS disease due to both *C. neoformans* and *C. gattii*, IRIS can manifest as hydrocephalus, new onset or enlarging CNS lesions, or the appearance of disease elsewhere e.g., lymph node enlargement and aseptic or relapsing meningitis. In SOT recipients, IRIS affects about 5–11% of patients, typically 4–6 weeks after antifungal therapy has commenced [138]. Tacrolimus, mycophenolate and prednisone are all associated with IRIS, and discontinuation of calcineurin inhibitors is independently associated with this entity [139].

A diagnosis of exclusion, it is always accompanied by negative microbiology results in the presence of apparently worsening disease. Management includes the workup to rule out disease relapse and continuation of antifungal therapy. Consideration should be given to delaying any planned reductions in immunosuppression. Guidelines recommend corticosteroids, especially if symptoms are severe or persistent, although their benefits are not well defined [90,118].

## 8. Conclusions

CNS cryptococcosis in non-HIV-infected individuals is well recognized and is likely to occur more frequently as patient risk groups expand. Epidemiological studies focusing on at risk patient groups are required to more fully determine predictors of disease and understand outcomes. Surveillance of both *C. neoformans* and *C. gattii* infection is also warranted to identify new host risk groups and virulence characteristics. Treatment remains challenging particularly among immunocompromised individuals who are more vulnerable to IRIS complications but also in non-transplant individuals who may require longer induction and total duration of therapy which may have to be tailored to the individual’s needs. Monitoring of response to therapy is essential to minimize the substantive morbidity from CNS cryptococcosis. There is a dearth of robust clinical trial data to inform management decisions, and guidelines have to rely heavily on expert opinion. Large collaborative networks will be required to recruit sufficient patients into trials with meaningful end-points to address some of the most pressing clinical questions. What are the optimal doses and durations of currently available anti-fungals for the different patient groups, and infecting organisms? What, if any, immune-modulatory approaches work for patients at different points of the inflammatory parabola? Clinicians urgently need answers to such basic questions, as we anticipate increasing patient numbers and realise that we should not simply rely on data from HIV-associated disease.

## Figures and Tables

**Table 1:** Proposed taxonomical changes for the *Cryptococcus neoformans* and *Cryptococcus gattii* species complex: nomenclature, genotype and geographic distribution of genotype.

Current Nomenclature	Genotype	Proposed Nomenclature	Geographical Region
*C. neoformans* var. *grubii*	VN1, VNII (serotype A)	*C. neoformans*	Worldwide
*C. neoformans* var. *neoformans*	VNIV (serotype D)	*C. deneoformans*	Europe (mainly) but can be found worldwide
*C. neoformans* inter-variety hybrid	VNIV (serotype AD)	*C. neoformans* X *C. deneoforamns* hybrid	Worldwide (uncommon)
*C. gattii*	VGI	*C. gattii*	Australia, PNG, Asia, USA, Mexico
	VGII	*C. deuterogattii*	British Columbia, Canada, Pacific North West USA, other regions USA, Asia, South America, Mexico
	VGIII	*C. bacillisporus*	USA, Asia, South America, Mexico
	VGIV	*C. teragattii*	**Africa**, Asia, South America, Mexico
	VGIV/VGIIIc	*C. decagattii*	Worldwide (rare)

Adapted from Hagen et al. [9]. Other hybrids: *C. neoformans* var. *neoformans* X *C. gattii* VGI hybrid to be assigned as *C. deneoformans* X *C. gattii* hybrid; *C. neoformans* var. *grubii* X *C. gattii* VGI hybrid to be assigned as *C. neoformans* X *C. gattii* hybrid; *C. neoformans* var. *grubii* X *C. gattii* VGII hybrid to be assigned as *C. deneoformans* X *C. deuterogattii* hybrid [9]. Bold font indicates predominant genotype in this region.

**Table 2:** Summary of main findings of patient details, clinical features, and clinical outcomes from cohorts of cryptococcosis including central nervous system infection in HIV-negative individuals cohorts (n > 20 patients).

	South Africa [47]	Australia and NZ [7]	USA [21]	Thailand [24]	Thailand, [48]	Vietnam [25]	Taiwan [37]	Taiwan [36]	USA [22]	China [23]	USA [18]	China [14]	USA [17]
**Cohort details**													
Restricted to meningitis	Yes	No	No	No	No	Yes	Yes	No	No	Yes	No	Yes	
Prospective	No	Yes	Yes	No	No	Yes	No	No	No	No	No	No	Yes
Multi-site	No	Yes	Yes	No	No	No	No	Yes	No	No	Yes	No	Yes
Time period	1991–1994	1994–1997	1990–1996	1987–2003	1996–2005	1998–2007	2000–2009	1997–2010	1996–2010	1998–2013	2004–2012	2000–2017	2013–2016
Number enrolled	21	200	306 *	37	29	57	51	149	194	106	1637	255	145
**Clinical features**													
Age (range)	38 (9–72)	NR	55 (1–84)	49 (16–83)	44 (16–83)	34 (15–75)	60 (31–88)	NR	55 (NR)	37 (NR)	58 (18–98)	39 (NR)	57 (17–89)
Male	71%	NR	61%	27%	31%	54%	63%	63%	61%	69%	NR	72%	66%
Headache	62%	NR	38% **	24%	NR	100%	64%	NR	41%	76%	35% $$	97%	51%
Fever	33%	NR	44%	57%	NR	77%	60%	NR	33%	63%	NR	82%	28%
Chest involved (imaging)	0%	60%	36%	74%	35%	13%	NA	NR	39%	NR	34%	NR	64%
**Lab features**													
ICP >20cm water (n/N)	NR	NR	NR ***	56% (5/9)	NR ###	NR	65% (24/51)	22% ## (32/65)	12% ## (24/194)	82% (87/106)	NR	76% (194/255)	NR $
**Immune status**													
Receiving immunosuppression	NR	14%	28%	41%	52%	12%	NR	NR	58%	14%	NR	15%	>47%
SOT	NR	6%	18%	0%	0%	0%	0%	2.7%	42%	<1%	10%	<1%	34%
Malignancy	NR	16%	18%	16%	21%	0%	28.6%	26%	19%	<1%	NR	3%	17%
Rheumatic disorders	NR	9.5%	13%	16% (SLE)	24% (SLE)	9%	18.4%	NR	4%	11%	NR	8%	16%
Apparently healthy	NR	31.1%	22%	22%	31%	81%	8.2%	15%	19%	NR	NR	36%	17%
**Prognosis**													
Case-fatality	9% #	NR	30%	27%	35%	19%	33%	35% @	14% @@	42%	34%	5% #	NR

Abbreviations: NR, not reported, and not possible to infer; NZ, New Zealand. * 291 ‘definite’ or ‘probable’ cryptococcosis; ** 73% of those w CNS disease; *** Median CSF pressure from 88 CNS patients was 23 cm H20; ### Median CSF pressure from six patients was 25.5 cm H20; ## Over 25cm H20; # Authors note issues with loss to follow up and patients going home to die; @ 10-week mortality; @@ 90 day mortality; $ But high incidence of refractory raised ICP indicated by 26 (18%) receiving therapeutic LPs and 8 (6%) undergoing surgical shunt; $$ ‘meningitis syndrome’.

**Table 3:** General recommendations for the antifungal treatment of central nervous system cryptococcosis due to *Cryptococcus neoformans* and *Cryptococcus gattii* in patients without human immunodeficiency virus infection (adapted from Perfect et al. [90] and Chen et al. [118]).

Host Risk Group or Clinical Setting	Preferred Antifungal Therapy	Alternative Antifungal Regimens	Duration of Therapy and Comments
***Cryptococcus neoformans* infection**			
Organ transplant patients	Induction therapy: L-AMB 3 mg/kg daily (or ABLC 5 mg/kg daily) plus 5-flucytosine 100 mg/kg daily, for 2 weeks Consolidation therapy: fluconazole 400–800 mg daily, for 8 weeks Maintenance therapy: fluconazole 200–400 mg daily, for 12 months	Induction therapy L-AMB 6 mg/kg daily or ABLC 5 mg/kg daily or AmB-D 0.7–1.0 mg/kg daily, for 4–6 weeks	The higher dose of L-AMB can be considered in cases high cryptococcal burden, or in presence of neurological complications OR where used alone without 5-flucytosine
Non-HIV infected, non-transplant hosts including, immunocompetent patients	Induction therapy: L-AMB 3 mg/kg daily (or AmB-D 0.7–1.0 mg/kg daily) plus 5-flucytosine 100 mg/kg daily, for ≥4–6 weeks Consolidation therapy: fluconazole 400–800 mg daily, for 8 weeks Maintenance therapy: fluconazole 200–400 mg daily, for 12 months	Induction therapy: L-AMB 3 mg/kg daily or AmB-D 0.7–1.0 mg/kg daily, for ≥6 weeks	Although the term ‘maintenance’ therapy is used, therapy aims to cure or eradicate infection, and longer durations of therapy may be required.
***Cryptococcus gattii* infection**			
All patients	Induction therapy: L-AMB 3 mg/kg daily (or AmB-D 0.7-1.0 mg/kg daily) plus 5-flucytosine 100 mg/kg daily, for at least 6 weeks Consolidation/maintenance therapy: fluconazole 400 daily, for 12–18 months	-	Induction therapy with fluconazole monotherapy is not recommended as there is a high probability of treatment failure [121]. Fluconazole at higher doses e.g., 800 mg daily may be used safely and with good efficacy. Longer total duration of therapy may be required. Surgical excision of mass lesions where appropriate

Abbreviations: ABLC, amphotericin B lipid complex; AmB-D, amphotericin B deoxycholate; L-AMB, liposomal amphotericin B.
